# Violent Asthmatic Fits, Occasioned by the Effluvia of Ipecacuanha

**Published:** 1810-09

**Authors:** William Scott


### *To the Editors of the Medical and Physical Journal.*

GENTLEMEN,

I Request you would have the goodness to give the following case, a place in your truly useful Journal, for the information of my much esteemed friend Dr. Hamilton, Mr. Royston, and Mr. Spencer. The patient was my mother, the person who treated and published the case was my father, well known in the republic of letters.

I am &c.

WALTER SCOTT, M. D.

*Stamfordham,*
*June 25,* 1810.

---

*Violent Asthmatic Fits, occasioned by the Effluvia of Ipecacuanha.* By WILLIAM SCOTT, M. D. V. Edin. Med. and Ph. Com. v. iv. p. 75.

Mrs. S———, of Stamfordham, Northumberland, married a medical gentleman in the year 1759, being then about 26 years of age. She had been always remarkably healthy before that period, and quite free from nervous or other complaints, except a trifling head-ach that used to affect her temples and forehead, sometimes for a night or two, about the time of menstruation.

The first year or two after her marriage she enjoyed her usual good health and spirits in general, but sometimes she was afflicted with a very troublesome shortness of breathing, attended with a remarkable stricture about the throat and breast, and with a particular kind of wheezing noise. These fits came on very suddenly, and without any exciting cause that at first could be assigned; they were often so violent as to threaten immediate suffocation; they lasted sometimes for a longer and sometimes for a shorter time, but generally went off in two or three days, with a spitting of a tough phlegm, which she said had a disagreeable metallic taste; during the absence of those paroxysms she enjoyed her usual good health and spirits; she had children, but suffered as little as any woman could do, either in breeding or lying-in; it was not observed that she was more subject to these fits when with child than at other times; blood was taken, and pectoral medicines given without any relief.

About

About a year and a half or two years after her marriage, she observed to me, that she had remarked these fits had always attacked her when exposed to the effluvia of ipecacuanha; this was looked on at first as a fancy, and little regard paid to it for some time. However, frequently after this, when any of that medicine was powdering, she immediately called out, perhaps from a different room, that she found the ipecacuanha, and that they would find her immediately affected by it. This, I and several others saw frequently happen, as she said; so that we were at last convinced, to a demonstration, that the effluvia of the medicine so affected her nerves, as to produce a remarkable degree of spasm about her throat and breast.

Having thus had ssveral repeated proofs of the effects the medicine had, great precaution was taken for several years to send her out of the house, whenever I had occasion to use ipecacuanha; by this precaution the bad effects were prevented, during which time she enjoyed perfect good health.

Betwixt nine and ten o'clock in the evening (June 3d, 1775), I had received a quantity of ipecacuanha; without considering, I opened it out, and put it into a bottle; my wife was near at the time, and then in perfect health; almost before it was quite put into the bottle, she called out that she felt the ipecacuanha affect her throat, on which she was immediately seized with stricture upon her breast, and difficulty of breathing. She was advised to walk into the air, to try if that would remove it, but it had little or no effect; she went to bed some little time afterwards; was extremely ill all night; at three o'clock next morning I saw her, when she was gasping for breath at a window; was as pale as death; her pulse scarcely to be felt, and seemed evidently to be in the utmost immediate danger of suffocation. She had seven or eight ounces of blood taken from her arm; her feet put into warm water; an anodyne draught with seven or eight drops of laudanum; she also took frequently a table spoonful of oil of almonds; none of these medicines seemed to have any effect. She continued much in the same way, with few or no intervals of ease till about nine o'clock in the morning, when, being in a manner almost exhausted, she fell into a kind of disturbed sleep; the difficulty of breathing, with a wheezing noise, still continuing, but little abated; she slept a short time. and got out of bed again about eleven o'clock that forenoon, her breathing being still very difficult, and her eyes looked red and a little inflamed; after she got up, she became came easier towards the afternoon, and it was then supposed that the spasms were removed. Dr. Brown, an eminent physician of Newcastle upon Tyne, being in the neighbourhood, called upon Mrs. S.; being informed of what had happened, said, he had known a case nearly similar from the same cause; hoped as she then was relieved, that the spasms would be removed. He recommended to her riding out as soon as she was able, and the bowels to be kept open; towards bed-time, the same evening, the difficulty of breathing returned, and continued all night; had flannel cloths wrung out of warm water applied to her feet, breast, and throat, with little or no advantage; blood was again taken, and had also a blister applied to the sternum, still continuing to take a spoonful of the oil of almonds; she had some sleep about nine in the morning, and continued in bed until twelve; she got up, and was again a little easier during the day, but at night the asthmatic paroxysms returned with great violence. The same scene was continued eight days and nights successively; that is, she was generally a little easier from about eleven o'clock in the forenoon till ten or eleven o'clock at night, when the difficulty of breathing always returned very violently. However, after eight days, she rested better at night, the asthmatic fits were neither so long nor so violent; and about fourteen days from the accident, they were almost entirely removed. Altho' she is now in very good health, she has not quite recovered her usual strength and colour. Besides the above medicines, she took small quantities of an emulsion of spermaceti with lac. ammoniacum; had a dose of cooling physic; rode, and walked out occasionally; had a few anodyne draughts, with eight drops of laudanum; it could not be observed that she received benefit from any of them, except from the oil of almonds. She had a flow of the menses four or five days after the accident, although it was then only about the middle of the usual period; she coughed up at times some small quantities of blood, and had also some mixed with her stools and urine.

The reason why the laudanum, the most effectual and universal antispasmodic, was used in such small quantities, was, that it was known before, that she could never bear above eight or nine drops of it, as the common dose used to affect her with violent sickness at stomach, giddiness and pain in her head, to so great a degree, that for some years past she neither would take, nor could I with safety administer a larger dose. At the time the accident happened she she was not with child, nor had she had any for some years before.

The above effects of ipecacuanha I believe very seldom happen, and, no doubt, arise from some peculiarity of constitution, and from the stimulus applied to the parts affected, producing in one patient asthma, and in another catarrhus. Medical writers, at least as far as I can recollect, seem to have taken little or no notice of its ever producing such an effect.

Mr. Leighton, a very eminent surgeon at Newcastle, told me, that the effluvia of ipecacuanha had the very same effect upon Mrs. Leighton; and that he had once in particular very nearly lost her, from having some of it powdered in his shop.

The ipecacuanha that had the above effects upon Mrs. S. was the common officinal ash coloured.

